# Patterns and Predictors of Insufficient Antenatal Care Utilization in Nigeria over a Decade: A Pooled Data Analysis Using Demographic and Health Surveys

**DOI:** 10.3390/ijerph17218261

**Published:** 2020-11-09

**Authors:** Ziad El-Khatib, Emmanuel Kolawole Odusina, Bishwajit Ghose, Sanni Yaya

**Affiliations:** 1 Department of Global Public Health, Karolinska Institutet, 171 77 Stockholm, Sweden; ziad.khatib@gmail.com; 2 World Health Programme, Université du Québec en Abitibi-Témiscamingue (UQAT), Québec, QC J9X 5E4, Canada; 3 Department of Demography and Social Statistics, Faculty of Social Sciences, Federal University Oye Ekiti, Oye-Ekiti, Ekiti State, Nigeria; kolaodusina@yahoo.co.uk; 4 School of International Development and Global Studies, University of Ottawa, Ottawa, ON K1N 6N5, Canada; brammaputram@gmail.com; 5 The George Institute for Global Health, Imperial College London, London SW7 2AZ, UK

**Keywords:** antenatal care, maternal health care utilization, global health, Nigeria demographic and health survey

## Abstract

This study investigated the patterns of antenatal care (ANC) utilization and insufficient use of ANC as well as its association with some proximate socio-demographic factors. This was a cross-sectional study using pooled data Nigeria Demographic and Health Surveys from years 2008, 2013 and 2018. Participants were 52,654 women of reproductive age who reported at least one birth in the five years preceding the surveys. The outcome variables were late attendance, first contact after first trimester and less than four antenatal visits using multivariable logistic regression analysis. The overall prevalence of late timing was 74.8% and that of insufficient ANC visits was 46.7%. In the multivariable regression analysis; type of residency, geo-political region, educational level, household size, use of contraceptives, distance to health service, exposure to the media and total number of children were found to be significantly associated with both late and insufficient ANC attendance. About half of the pregnant women failed to meet the recommendation of four ANC visits. Investing on programs to improve women’s socio-economic status, addressing the inequities between urban and rural areas of Nigeria in regard to service utilization, and controlling higher fertility rates may facilitate the promotion of ANC service utilization in Nigeria.

## 1. Introduction

Despite several efforts by governmental and non-governmental agencies to reduce maternal mortality, maternal deaths remain high globally, particularly in low and middle-income countries (LMIC). Worldwide, about 830 women die from pregnancy or childbirth-related complications every day, and out of the estimated 303,000 maternal deaths in 2015, about 99 percent occurred in LMIC [1]. Sub-Saharan Africa has the highest mortality or morbidity due to reproductive ill-health among pregnant women [2]. The estimated maternal mortality ratio (MMR) in sub-Saharan Africa in 2015 was 547 per 100,000 live births [1]. With an estimated MMR of 814 per 100,000 live births, Nigeria remains the fourth highest contributor to the region’s maternal deaths and accounts for 19 percent globally [3,4]. Nigeria consists of two percent of the world’s population, but contributes about 10 percent of the worldwide estimates of maternal deaths [5]. In Nigeria, maternal mortality is still relatively high compared to what is obtainable in developed countries, though the ratio reduced to 814 in the year 2015 from 1350 in the year 1990 [3]. In addition, the 2018 Nigeria demographic and health survey reported 512 deaths per 100,000 live births for the seven year period preceding the survey [6].

The Nigerian health care system is poorly developed and health care access is heavily based on out-of-pocket payments. Health resources (for example health centers, personnel, and medical equipment) are inadequate across the country, particularly in rural areas [7]. The Nigerian government established the National Health Insurance Scheme (NHIS) with the aim of improving access to health care and reducing the financial burden of out-of-pocket payments for health care services, but unfortunately a significant proportion of the population, including women, are left out and do not benefit from it [8]. Harrison (1997) highlighted the worsening scenario of maternal mortality in Nigeria subsequent to, and despite, the launch of the Safe Motherhood initiative in 1987. This article focused on three fundamental, although broad, factors underlying the devastating trend in maternal mortality, particularly in Nigeria [1]. These factors are not isolated events that affect maternal mortality but are rather interlinked with several other factors leading to a given trend/result. One research article has shown that poverty, in the absence of appropriate health insurance, leads to the inability of women to seek proper medical care and attention leading to an increase in maternal mortality [3]. Important issues in the efforts towards the reduction of maternal and childhood mortality include the use of maternal health care services [9]. In view of the high level of maternal mortality in Nigeria, despite the efforts of government and non-government institutions, there is a need for more evidence-based research to unveil the factors affecting the use of antenatal care services. Globally, to achieve the third sustainable development goal of reducing maternal deaths to less than 70 per 100,000 live births by 2030, requires more studies [10,11] on effective interventions [9]. Therefore, knowledge and proper understanding of access and demand side factors are important to inform policy. The pertinent question relates to the identification of factors that have contributed to this high maternal mortality and establish whether insufficient antenatal care is one of the factors.

At least 40% of women in LMIC do not receive antenatal care during pregnancy [9]. Whereas utilization of recommended antenatal care has been associated with the reduction of maternal morbidities and mortalities [10]. As a key component of maternal health care services, the timing of antenatal care (ANC) and the numbers of ANC visits are very vital [6]. The use of health facilities has been significantly associated with ANC visits and also sufficient ANC involves both the use of services and the sufficiency of the content within the services [9]. The 2018 Nigeria demographic and health survey reports that only eighteen percent of women had their first ANC visits in the first trimester, which calls for more evidence-based research and approaches to enhance ANC attendance [6].

Similarly, literacy is linked to women’s empowerment, age at marriage and ability to seek health care services [12]. Regarding some of these interlinked factors and following multiple summits and conferences, the United Nations established eight millennium development goals (MDGs), expected to be achieved by 2015. The establishment of the sustainable development goals (SDGs) further underpins the importance of the above interlinked factors. The point underpinning the current study is the relative dearth of studies on the patterns of and predictors of insufficient antenatal care utilization.

Nigeria adhered to WHO’s focused/goal oriented approach or model, also known as the basic ANC model, which includes four ANC visits and was introduced in 2002 for evidence-based intervention for healthy pregnant women [13]. This is designed to compute and explain ANC utilization to policy makers as well as compare countries and monitor ANC coverage. Rationalizing further, WHO stressed the importance of antenatal care towards reducing maternal mortality. Again, several research articles have stressed the importance of “optimal” antenatal care, which acts as a “cornerstone” probably through immunization programs and iron prophylaxis as well as timely identification of pregnancy related risks and complications [14,15,16]. These factors help alleviate maternal mortality and morbidity and reinstate harmonious relationships between pregnant mothers and caregivers including health care provider(s).

Even the earlier introduced Safe Motherhood initiative focused on two crucial strategies, namely improving antenatal care and training birth attendants to reduce maternal mortality [17]. However, evidence has revealed that 70% of the indirect causes of maternal deaths were due to pre-existing disorders exacerbated by pregnancy [18]; and therefore, the role of antenatal care becomes even more critical. Accordingly, the role of antenatal care in reducing maternal mortality and morbidity is evidence-based [19], although it should be noted that there are research articles that debate the importance of antenatal care [20,21]. Additionally, it has been noted that there are disparities in the provision of antenatal care and use, not only inter-country but also inter-region [15,19]. Such disparities and the scarcity of research studies investigating the trend of antenatal care utilization further support the current study. In addition, a number of studies support the fact that both the demand and supply side factors play a role in ANC attendance [22,23,24,25]. It is beyond the scope of the current study to incorporate all of these factors. However, subject to the limitations of data availability, an attempt has been made to incorporate factors that are related to ANC utilization. These do not only aid the comprehension of ANC trends in Nigeria but also provide valuable policy inputs. Therefore, the aim of the study is to investigate the patterns of and predictors of insufficient ANC utilization in Nigeria.

## 2. Materials and Methods

Data for this study were derived from three rounds of demographic and health surveys (DHS) in Nigeria, which provided information on maternal and child health. In Nigeria, the surveys are implemented by the National Population Commission (NPC) with the financial and technical assistance of Inner-City Fund (ICF) through the United States Agency for International Development (USAID)-funded DHS program. DHS surveys collect nationally representative information on a wide range of public health related topics such as anthropometric, demographic and socioeconomic information, family planning and domestic violence. The survey covered men and women aged between 15–49 years and children under 5 residing in non-institutional settings. For sampling, a three-staged stratified cluster design was employed, which was based on a list of enumeration areas (EAs) from the 2006 population census of the Federal Republic of Nigeria. The EAs are systematically selected units from the localities, which constitute the local government areas (LGAs). LGAs are subdivisions of each of the 36 administrative states (including the Federal Capital Territory called Abuja) and are classified under six developmental zones in the country. EAs were used to form the survey clusters called primary sampling units. A more detailed version of the survey was published elsewhere [26].

Nigeria is located in the West African sub-region. The country is made up of six regions. Though the most populous country in Africa, it is ravaged by high rates of maternal mortality, mortality of children under 5 years of age, low contraceptive usage, reproductive tract infections, and other maternal morbidity. The health sector generally is characterized by wide regional disparities in status, service delivery, and resource availability. The three tiers of the government in Nigeria (called federal, state and local) share responsibilities for providing health services and programs. Only women who became pregnant or experienced childbirth are eligible to utilize pregnancy care services and were included in this study.

Outcome variables were timing and inadequacy of ANC visits. This was categorized as: (1) first visit after the first trimester, and (2) insufficient ANC visits (less than 4 ANC visits). The timing of an ANC visit was calculated based on the criteria of initiation of the first visit within the first four months of gestation and subsequent visits by the fifth or sixth month [27].

For insufficient ANC visits, the WHO guidelines were used [28,29]—making at least four visits (yes/no). Explanatory variables were based on a broad literature review, and availability of the datasets, and the following variables were included in the analysis: year (2008, 2013 and 2018); age groups (15–19, 20–24, 25–29, 30–34, 35–39, 40–45, 45–49); type of place of residence (urban/rural); region (North Central, North East, North, West, South East, South South, South West); education (none, primary, secondary, post-secondary); wealth index (this procedure assigned scores and standardized the wealth indicator variables such as floor type, walls, roof, water source, sanitation facilities, radio, electricity, television, refrigerator, cooking fuel, furniture, number of persons per room); husband’s education (no education, primary, secondary, higher); sex of household head (male/female); total number of children born per household (1–2, 3–4, >4); health insurance (yes/no); use of contraceptives (not using, using); employment status (not working, working); distance to health services (big problem, not a big problem); household size (less than 5, 5+); marriage type (monogamy, polygyny); decision making on health (does not participate, participates) and exposure to the media (yes/no). These variables were selected based on the reports from previous studies [2,11,16,18,22,23,24,25,26,27,29,30,31,32,33,34]. Results are displayed in Table 1.

Participants were 52,654 women aged between 15–49 years reporting at least one birth in the five years preceding the surveys. The outcome variables were late attendance (first contact after first trimester) and less than four antenatal visits. The dichotomous nature of each of the categories of the outcome variables informed the use of multivariable logistic regression analysis. Sample weights were divided by 3, applied and used appropriately. Stata version 14 was used to merge datasets, recode variables where necessary and for data analysis. The study considered and used the World Health Organization guidelines, which were published in 2002 and recommended a minimum of 4 ANC visits.

The datasets, which were nationally representative, were from the Nigeria demographic and health surveys. The external validity of the study was enhanced by the standard procedure, the sampling method of DHS and the sample size—which was sufficiently large. Datasets from three surveys (2008, 2013, 2018) were merged into one to perform pooled analysis. Pooled analysis enhances statistical power and the capacity to compare results or outcomes across different periods of time or years. Pooled analysis enhances greater variations in participants, treatments, and subgroup analyses in the pooled dataset. To adjust for the cluster sampling technique of the surveys, we used complex survey modules for all analysis by accounting for primary sampling units, sample strata and sample weight. Following that, descriptive analyses were carried out to calculate the prevalence rates of insufficient utilization of ANC. Chi-square tests were performed to examine the bi-variate association between insufficient ANC and the socio-demographic variables. Variables that were found to be significant at alpha 5% were entered into the multiple regression analysis [35]. Binary logistic regression analyses were carried out to calculate the odds ratios of the association between two outcome measures with the socio-demographic variables. The level of significance was set at alpha 5% for the regression model. Computation of wealth index was obtained through DHS using principal components analysis (PCA) to assign the wealth indicator weights. Thereafter, the factor coefficient scores (factor loadings), and z-scores were calculated. Finally, for each household, the indicator values were multiplied by the loadings and summed to produce the household’s wealth index value. The standardized z-score was used to disentangle the overall assigned scores and to classify the scores as poorest, poorer, middle, richer, and richest.

The analyses were done using publicly available data from demographic health surveys. Ethical procedures were the responsibility of the institutions that commissioned, funded, or managed the surveys. All DHS surveys have been approved by ICF International as well as an institutional review board (IRB) in Nigeria to ensure that the protocols are in compliance with the U.S. Department of Health and Human Services regulations for the protection of human subjects (http://dhsprogram).

## 3. Results

### 3.1. Socio-Demographic Characteristics

Table 2 summarizes the socio-demographic description of the participants. Mean age was 29.6. Most of the respondents were: adherents of Islam (61.9%), living in rural areas (65.2%), working (67.8%), belonged to a monogamous family type (68.3%), did not participate in health decision making (61.0%), had access to health facility (67.5%), did not use contraceptives (88.9%), from male-headed households (92.5%), had no health insurance (98.2%), not exposed to the media/family planning information through the media (65.0%) and about half had no formal education (48.1%).

### 3.2. Timing of ANC Visits

**Figure 1:**
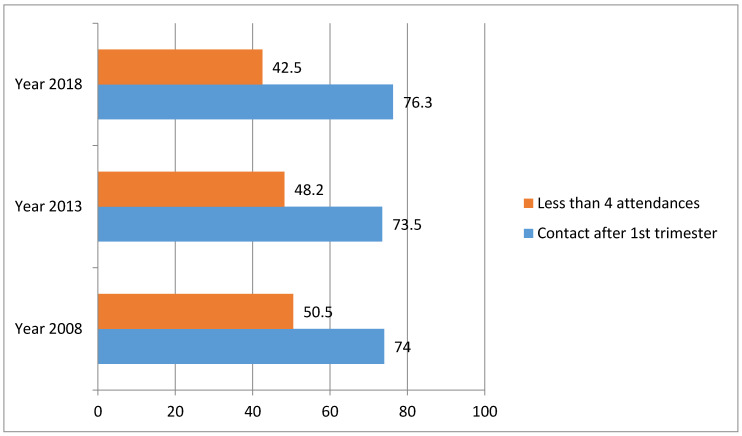
Patterns of insufficient antenatal care. Utilization in Nigeria.

Overall, out of the 52,654 respondents, 24,576 attended less than four ANC visits while out of 35,300 women who sought ANC, 26,407 went after the first trimester. As shown in Table 2, the overall prevalence of late timing was 74.8% (95% CI = 74.4–75.3). The prevalence of women who attended less than a minimum of four ANC visits was 46.7% (95% CI = 46.2–47.1). While there had been a marginal decrease in the prevalence of late ANC visits between 2008 and 2013, an increase was observed between 2013 and 2018. A decrease in the prevalence of women who attended less than a minimum of four ANC visits was observed between 2008 and 2013, and also between 2013 and 2018. Details on the timing of ANC visits are presented in Figure 1.

The table also indicates that the rate of late contact was higher among women who were: between 25–29 years of age, residents of rural and northwest regions, followers of Islam, had formal education, lived in richer households, husbands had secondary education, working, belonged to a monogamous marriage type, had >4 household members, did not participate in making decisions on health, had problems accessing health services, did not use contraceptives, exposed to the media, had >4 children, male headed households and had no health insurance while higher patterns were observed for insufficient ANC visits among women 25–29 years of age, residents of rural and northwest regions, followers of Islam, with no formal education, working, belonged to monogamous marriage type, had >4 household members, did not participate in making decisions on health, had problem accessing health services, did not use contraceptives, not exposed to the media, belonged to poorest household category, husbands had no formal education, male headed households, had >4 children and had no health insurance.

### 3.3. Odds of Late ANC Contacts

The results of multivariable regression analysis (Table 3) on the association between utilization of ANC and the socio-demographic variables are summarized in Table 3. The odds ratios of late contacts were significantly higher among women in the North East (OR 1.68; 95% CI 1.55–1.82), South South (OR 2.00; 95% CI 1.82–2.20), South East (OR 1.37; 95% CI 1.24–1.50), South West (OR 1.67; 95% CI 1.53–1.81), and North West (OR 2.97; 95% CI 2.70–3.25) compared with North Central; among those who had more than four children (OR 1.27; 95% CI 1.16–1.39) or 3–4 children (OR 1.16; 95% CI 1.08–1.24) compared with those who had 1–2 children, among female headed households (OR 1.11; 95% CI 1.02–1.21) compared with those households headed by a male; among households with more than four members (OR 1.19; 95% CI 1.12–1.27) compared with households with four/less than four members and lower in the rural (OR 0.79; 95% CI 0.74–0.84) areas compared with urban areas, among those who had secondary education (OR 0.92; 95% CI 0.84–1.00) and post-secondary education (OR 0.76; 95% CI 0.67–0.86) compared with those with no formal education; among those who claimed their husbands had secondary education (OR 0.90; 95% CI 0.82–0.98) and post-secondary education (higher) (OR 0.84; 95% CI 0.76–0.93) compared with women who claimed their husbands had no formal education; among those who were working (OR 0.89; 95% CI 0.84–0.95) compared with women who were not working, among women who claimed distance was a big problem to accessing health facilities (OR 0.93; 95% CI 0.88–0.99) compared with women who claimed distance was not a big problem and among women who were using contraceptives (OR 0.92; 95% CI 0.86–0.98) compared to those who were not using contraceptives.

### 3.4. Odds of Less than Four ANC Visits

In general, the odds ratios of less than a minimum of four visits were higher among rural residences (OR 1.23; 95% CI 1.16–1.30) compared with urban residences, among women in the North West (OR 1.73; 95% CI 1.62–1.86) and South South (OR 1.62; 95% CI 1.48–1.76) compared with North Central, among those who had more than four children (OR 1.30; 95% CI 1.20–1.40) or 3–4 children (OR 1.19; 95% CI 1.12–1.27) compared with those who had 1–2 children and among households with five or more members (OR 1.07; 95% CI 1.01–1.13) compared with households with less than five members while the odds ratios of less than a minimum of four visits were lower among those in the older age groups (20–24 (OR 0.89; 95% CI 0.81–0.99), 25–29 (OR 0.81; 95% CI 0.73–0.90), 30–34 (OR 0.68; 95% CI 0.61–0.76); 35–39 (OR 0.69; 95% CI 0.61–0.78), 45–49 (OR 0.69; 95% CI 0.59–0.81) and 40–44 (OR 0.66; 95% CI 0.58–0.75)) compared with those in the youngest age group (15–19 years). The associations of less than four ANC visits were negative in some regions (South West (OR 0.39; 95% CI 0.36–0.44) and South East (OR 0.54; 95% CI 0.48,–0.60)), educational status among the respondents (primary (OR 0.66; 95% CI 0.62–0.70), secondary (OR 0.54; 95% CI 0.50–0.58) and post-secondary education (OR 0.34; 95% CI 0.29–0.40)), and husband’s education (primary (OR 0.59; 95% CI 0.55–0.62), secondary (OR 0.56; 95% CI 0.52–0.59), higher (OR 0.49; 95% CI 0.45–0.54)), household wealth status (poorer (OR 0.69; 95% CI 0.64–0.73), middle (OR 0.49; 95% CI 0.46–0.53), richer (OR 0.42; 95% CI 0.38–0.45) and richest (OR 0.27; 95% CI 0.24–0.31)), employment status (working (OR 0.82; 95% CI 0.78–0.86)), having health insurance (OR 0.68; 95% CI 0.55–0.85), health decision making (OR 0.82; 95% CI 0.78–0.86), having exposure to the media (OR 0.68; 95% CI 0.65–0.71) and using contraceptives (OR 0.67; 95% CI 0.62–0.72).

## 4. Discussion

The study considered datasets from three Nigeria demographic and health surveys (2008, 2013 and 2018). The datasets were merged into one to perform pooled analysis. Unlike several other related studies [4,9,10,19,29,30,31,33,36], this study enjoyed the benefits of pooled analyses. Pooled analysis enhanced variations in participants, treatments, statistical power, and comparison of outcomes across different years. There was little or no reduction in the late ANC visits and insufficient attendance over time, 2008 to 2018. The evidence revealed an association between the increased use of pre-natal health care services and the socio-economic status of women [37]. Overall, the majority of the participants reported late ANC visits and also insufficient attendance. A marginal decrease was observed in late ANC visits between 2008 and 2013 but an increase was observed between 2013 and 2018 while a decrease was observed with respect to insufficient attendance between 2008 and 2018.

The late ANC visits and insufficient attendance are serious issues of concern in Nigeria. Socio-economic factors of women play a significant role in determining the utilization of antenatal health care services. Intervention policies and programs should address socio-economic vulnerabilities to reduce under-utilization of antenatal care services [5,37,38]. The poor attendance might be connected with the high maternal and child mortalities in the country [39]. A large percentage of the maternal mortality in sub-Saharan countries [11] stemmed from Nigeria. With the high percentage of late ANC visits and insufficient ANC attendance, much needed to be achieved in Nigeria to achieve the third sustainable development goal, that is, good health and well-being for all [40]. With more studies (on determinants of access and demand, as people have to access facility before they enjoy its content), health facilities and various intervention programs, a reduction in late ANC visits and insufficient attendance is expected.

The older women were more sensitive to ANC utilization. The likelihood of insufficient ANC visits was more common among the younger cohorts of women when compared with the older age group. A similar pattern was observed for ANC visits after the first trimester (late ANC visit) among the women. Those living in rural areas, compared to urban areas, were less likely to make at least four ANC visits but more likely to avoid attending ANC visits late in the first trimester. Many of the health facilities in Nigeria are situated in urban areas and they are accessible. Effective policies to address rural–urban differentials in this regard is a requirement for better ANC health care services [41]. In the same vein, regional disparities were noted in the underutilization of ANC services. Disparities in the underutilization of ANC services might be connected with regional disparities in socio-demographic factors such as education, wealth status, age and employment [37]. Improvement in social services through socio-economic and health policy interventions may help to reduce regional disparities in the underutilization of ANC in Nigeria [38].

In addition to the level of secondary or higher education of the women [40], their husband’s level of secondary or higher education can also influence the likelihood of late ANC attendance [29] in the first trimester of pregnancy as well as less than four ANC visits [5]. Therefore, investment in female education may enhance early initiation of ANC visits [37]. Education affords women better access to health care information and the ability to participate in decision making about health care services and also the capacity to understand the negative consequences of not utilizing prenatal health care services [37,42]. For wealth index quintiles comparison with the poorest quintile, the higher the wealth index quintile, the lower the likelihood of less than four ANC visits. This finding is consistent with findings from similar previous studies on the economic status of women and its positive influence on prenatal care service utilization [37,43,44]. It should be noted, however, that the findings for education level, region of residence and wealth index quintiles were not monotonic. Findings from this study reveal that the increasing level of education from primary to secondary, for example, decreases the relative likelihood of insufficient ANC utilization. Several research studies have noted multiple factors that play a role in ANC utilization and the most important of these are residence [32,36], regional disparities, economic disparities and education level [5,32,37]. These findings are consistent across multiple studies and across multiple countries [27,34,45,46,47,48,49].

As discussed above, employment status, number of children, distance to health service [5,50] and use of contraceptives also influenced ANC utilization. In addition, having health insurance influenced the frequency of ANC visits. Health insurance reduced out-of-pocket payments for drugs and other ANC services and thus encouraged ANC attendance. Exposure to family planning information through the media afforded the women the opportunity to receive information and counselling about the need for ANC utilization. This explained the association found between exposure to the media and ANC utilization. These findings are again consistent with previous research studies [37,51,52,53,54]. Overall, women in Nigeria have not achieved the old WHO guideline of a minimum of four ANC visits. Achievement of the new WHO guideline of a minimum of eight ANC visits may be a mirage in the short term. Therefore, to improve the use of ANC and reduce the negative outcomes, such as maternal morbidity and mortality, program administrators and policy makers should address socio-demographic vulnerabilities of women.

This study included a large sample from three representative surveys to measure the prevalence of late and insufficient ANC visits among women aged 15–49 years. Data were analyzed taking into consideration the cluster effects to ensure the precision of the estimates. Women from both urban and rural areas were included, across all six regions, which increases the external validity of the findings. Nonetheless, the surveys were cross-sectional and hence the associations do not equate to causations. The study did not consider the new WHO guideline of a minimum of eight ANC visits because two out of the three waves of the datasets used (2008 and 2013 Nigeria demographic and health surveys) were released before the new recommendation and for ease of comparison with similar studies. The study addressed only ANC utilization and not quality of care received. There is a need for further qualitative study to deepen knowledge and understanding of barriers to ANC uptake. In addition, identification of more successful interventions through implementation research may help to improve the uptake of ANC.

## 5. Conclusions

Based on the analysis of three rounds of DHS surveys, this study shows that the prevalence of late timing of ANC and insufficient ANC attendance remained high despite the different efforts to ensure prompt and sufficient attendance. The high occurrence of late timing and insufficient attendance threatens health development in Nigeria and the achievement of the sustainable development goals for maternal and child health. To reduce maternal and child morbidity and mortality, policy makers should make efforts to improve prompt and timely ANC attendance among women in Nigeria. Furthermore, lower educational level, residence, geographical region, household size, use of contraceptives, poor household wealth status, and having more than two children also appeared to be associated with insufficient utilization of ANC services. These findings highlight the need for investing in improving women’s socio-economic status as a potential strategy to promote the utilization of ANC services in the country.

## Figures and Tables

**Table 1:** Operational definition of variables used for analysis.

Study Variables	Operational Definition and Coding
Year	1 = 2008, 2 = 2013, 3 = 2018
Age	Self-reported ages coded as 1 = 15–19, 2 = 20–24, 3 = 25–29, 4 = 30–34, 5 = 35–39, 6 = 40–44, 7 = 45–49
Regions	Regions of respondents as provided by NDHS *, 1 = North Central, 2 = North East, 3 = North West, 4 = South South, 5 = South East and 6 = South West
Type of place of residence	1 = Urban, 2 = Rural
Education	Self-reported 1 = None, 2 = primary, 3 = secondary, 4 = Post-secondary
Wealth Index	According to household index classified into 5 categories by NDHS, 1 = Poorest, 2 = Poorer, 3 = Middle, 4 = Richer and Richest
Husbands education	1 = No education, 2 = Primary education, 3 = Secondary education and 4 = Higher
Sex of household head	1 = Male, 2 = Female
Total children born	1 = 1–2, 2 = 3–4 and 3 = >4
Has health insurance	1= No, 2 = Yes
Distance from health services	1 = Big problem, 2 = Not a big problem
Religion	1 = Christianity, 2 = Islam, 3 = others
Use of contraceptive	1 = Not Using, 2 = Using
Employment status	1 = Not working, 2 = Working
Household size	Less than 5, 5+
Decision making on health	1 = Not participate, 2 = participate
Expose to mass media	0 = No, 1 = Yes

* NDHS: Nigeria Demographic and Health Survey.

**Table 2:** Socio-demographic profile of the sample population, socio-demographic patterns in the uptake of antenatal care (ANC) services, Nigeria Demographic and Health Survey (NDHS) 2008–2018.

Variables	*N* (52,654)	%	Contact after 1st Trimester (*n* = 26,407) 74.8% (95% CI = 74.4, 75.3)	*p*-Value	Less than 4 Attendances (*n* = 24,576) 46.7% (95% CI = 46.2, 47.1)	*p*-Value
Year			% (95% CI)		% (95% CI)	
2008	14,475	27.5	74.0 (73.1–74.9)		50.5 (49.7–51.3)	
2013	18,214	34.6	73.5 (72.7–74.3)	0.001	48.2 (47.4–48.9)	0.001
2018	19,964	37.9	76.3 (75.6–76.9)		42.5 (41.8–43.2)	
Age groups						
15–19	3060	5.8	4.6 (4.4–4.9)		07.7 (07.4–08.0)	
20–24	9936	18.9	17.8 (17.3–18.3)	0.009	20.6 (20.1–21.2)	0.001
25–29	13,913	26.4	27.0 (26.5–27.6)		25.6 (25.0–26.1)	
30–34	11,285	21.4	22.3 (21.8–22.8)		18.7 (18.2–19.2)	
35–39	8423	16.0	16.8 (16.3–17.2)		14.9 (14.4–15.3)	
40–44	4257	8.1	8.3 (8.0–8.6)		08.4 (08.0–8.7)	
45–49	1781	3.4	3.2 (3.0–3.4)		04.1 (03.9–04.4)	
Religion						
Christian	19,522	37.1	46.0 (45.4–46.6)		22.9 (22.4–23.4)	
Islam	32,600	61.9	53.1 (52.5–53.7)	0.001	75.4 (74.9–75.9)	0.001
Others	532	1.0	0.9 (0.8–1.0)		01.7 (01.5–01.9)	
Type of place of residence						
Urban	18,350	34.8	41.8 (41.2–42.4)		15.7 (15.2–16.1)	
Rural	34,304	65.2	58.2 (57.6–58.8)	0.992	84.3 (83.9–84.8)	0.001
Region						
North Central	7484	14.2	16.7 (16.3–17.2)		15.0 (14.6–15.5)	
North East	9237	17.5	20.7 (20.2–21.2)	0.001	27.4 (26.8 –27.9)	0.001
North West	18,786	35.7	23.7 (23.2–24.2)		44.6 (43.9–45.2)	
South East	4330	8.2	11.1 (10.8–11.5)		02.9 (02.7–03.1)	
South South	4778	9.1	11.1 (10.7–11.5)		07.4 (07.1–07.7)	
South West	8040	15.3	16.7 (16.2–17.1)		02.8 (02.6–03.0)	
Education						
None	25,328	48.1	34.7 (34.2–35.3)		71.8 (71.3–72.4)	
Primary	9576	18.2	22.4 (21.9–23.0)	0.001	15.3 (14.9–15.8)	0.001
Secondary	14,053	26.7	34.2 (33.6–34.8)		11.8 (11.4–12.2)	
Post-secondary	3697	7.0	8.6 (8.3–9.0)		01.0 (00.9–01.2)	
Wealth index						
Poorest	12,475	23.7	14.8 (14.4–15.2)		40.5 (39.9–41.1)	
Poorer	11,860	22.5	19.7 (19.3–20.2)	0.001	30.0 (29.5–30.6)	0.001
Middle	9965	18.9	22.4 (21.9–22.9)		16.9 (16.4–17.4)	
Richer	9305	17.7	23.3 (22.8–23.8)		09.3 (09.0–09.7)	
Richest	9048	17.2	19.8 (19.3–20.3)		03.2 (03.0–03.5)	
Employment Status						
Not working	16,941	32.2	28.4 (27.8–28.9)		40.6 (39.9–41.2)	
Working	35,713	67.8	71.6 (71.1–72.2)	0.001	59.4 (58.8–60.1)	0.001
Husbands education						
None	20,350	38.6	25.3 (24.8–25.8)		60.5 (59.9–61.1)	
Primary	9229	17.5	20.0 (19.6–20.5)	0.001	15.8 (15.3–16.2)	0.001
Secondary	16,118	30.6	37.5 (36.9–38.0)		18.5 (18.0–19.0	
Higher	6957	13.2	17.2 (16.8–17.7)		05.2 (05.0–05.5)	
Marriage type						
Monogamy	35,985	68.3	71.8 (71.3–72.4)		60.0 (59.4–60.6)	
Polygyny	16,669	31.7	28.2 (27.6–28.7)	0.001	40.0 (39.4–40.6)	0.001
Sex of household head						
Male	48,679	92.5	90.7 (90.3–91.3)		94.6 (94.3–94.9)	
Female	3975	7.5	9.3 (8.6–9.7)	0.062	05.4 (05.1–05.7)	0.001
Total children ever born						
1–2	17,371	33.0	32.8 (32.2–33.4)		28.2 (27.6–28.7)	
3–4	15,124	28.7	29.8 (29.3–30.4)	0.001	26.7 (26.2–27.3)	0.001
>4	20,159	38.3	37.4 (36.8–38.0)		45.1 (44.5–45.7)	
Household Size						
Less than 5	22,952	43.6	44.1 (43.5–44.7)		36.3 (35.7–36.9)	
5+	29,702	56.4	55.9 (55.3–56.5)	0.062	63.7 (63.1–64.3)	0.001
Health Decision Making						
Not participate	32,115	61.0	54.5 (53.9–55.1)		74.3 (73.8–74.8)	
Participate	20,539	39.0	45.5 (44.9–46.1)	0.001	25.7 (25.2–26.2)	0.001
Distance to health service						
Big problem	17,113	32.5	26.0 (25.4–26.5)		45.3 (44.7–46.0)	
Not a big problem	35,541	67.5	74.0 (73.5–74.6)	0.027	54.7 (54.0–55.3)	0.001
Use of contraceptive						
Not Using	46,824	88.9	86.2 (85.8–86.7)		95.6 (95.3–95.8)	
Using	5830	11.1	13.8 (13.3–14.2)	0.001	04.4 (04.2–04.7)	0.001
Has health insurance						
No	51,716	98.2	97.6 (97.4–97.8)		99.5 (99.4–99.6)	
Yes	938	1.8	2.4 (2.2–2.6)	0.001	00.5 (00.4–00.6)	0.001
Exposure to Media						
No	34,254	65.0	59.4 (58.8–60.0)	0.001	81.6 (81.1–82.0)	0.001
Yes	18,400	35.0	40.6 (40.0–41.2)		18.4 (18.0–18.9)	

**Table 3:** Predictors of inadequate use of ANC services in Nigeria, NDHS 2008–2018.

Variables	Contact After 1st Trimester (*n* = 26,407)	Less than Four Visits (*n* = 24,576)
Year	OR (95% CI)	OR (95% CI)
2008	1 (Ref)	1 (Ref)
2013	0.90 (0.85–97) **	0.82 (0.78–0.87) ***
2018	1.05 (0.99–1.12)	0.72 (0.68–0.76) ***
Age Groups		
15–19	1 (Ref)	1 (Ref)
20–24	0.93 (0.81–1.06)	0.89 (0.81–0.99) *
25–29	0.95 (0.83–1.09)	0.81 (0.73–0.90) ***
30–34	0.88 (0.76–1.02)	0.68 (0.61–0.76) ***
35–39	0.87 (0.75–1.02)	0.69 (0.61–0.78) ***
40–44	0.85 (0.72–1.01)	0.66 (0.58–0.75) ***
45–49	0.89 (0.72–1.10)	0.69 (0.59–0.81) ***
Religion		
Christian	1 (Ref)	1 (Ref)
Islam	1.17 (1.10–1.26) ***	0.98 (0.91–1.05)
Others	1.22 (0.93–1.62)	1.52 (1.25–1.84) ***
Type of place of residence		
Urban	1 (Ref)	1 (Ref)
Rural	0.79 (0.74–0.84) ***	1.23 (1.16–1.30) ***
Region		
North Central	1 (Ref)	1 (Ref)
North East	1.68 (1.55–1.82) ***	1.01 (0.94–1.08)
North West	2.97 (2.70–3.25) ***	1.73 (1.62–1.86) ***
South East	1.37 (1.24–1.50) ***	0.54 (0.48–0.60) ***
South South	2.00 (1.82 = 2.20) ***	1.62 (1.48–1.76) ***
South West	1.67 (1.53–1.81) ***	0.39 (0.36–0.44) ***
Education		
None	1 (Ref)	1 (Ref)
Primary	1.00 (0.92–1.09)	0.66 (0.62–0.70) ***
Secondary	0.92 (0.84–1.00) *	0.54 (0.50–0.58) ***
Post-secondary	0.76 (0.67–0.86) ***	0.34 (0.29–0.40) ***
Wealth index		
Poorest	1 (Ref)	1 (Ref)
Poorer	0.98 (0.89–1.07)	0.69 (0.64–0.73) ***
Middle	0.98 (0.88–1.08)	0.49 (0.46–0.53) ***
Richer	1.05 (0.95–1.17)	0.42 (0.38–0.45) ***
Richest	0.88 (0.78–0.99) *	0.27 (0.24–0.31) ***
Employment Status		
Not working	1 (Ref)	1 (Ref)
Working	0.89 (0.84–0.95) ***	0.82 (0.78–0.86) ***
Husbands education		
None	1 (Ref)	1 (Ref)
Primary	0.96 (0.88–1.05)	0.59 (0.55–0.62) ***
Secondary	0.90 (0.82–0.98) *	0.56 (0.52–0.59) ***
Higher	0.84 (0.76–0.93) **	0.49 (0.45–0.54) ***
Marriage type		
Monogamy	1 (Ref)	1 (Ref)
Polygyny	0.96 (0.90–1.02)	1.04 (0.99–1.09)
Sex of household head		
Male	1 (Ref)	1 (Ref)
Female	1.11 (1.02–1.21) *	0.95 (0.87–1.03)
Total children ever born		
1–2	1 (Ref)	1 (Ref)
3–4	1.16 (1.08–1.24) ***	1.19 (1.12–1.27) ***
>4	1.27 (1.16–1.39) ***	1.30 (1.0–1.40) ***
Household Size		
Less than 5	1 (Ref)	1 (Ref)
5+	1.19 (1.12–1.27) ***	1.07 (1.01–1.13) *
Health Decision Making		
Not participate	1 (Ref)	1 (Ref)
Participate	0.96 (0.91–1.02)	0.82 (0.78–0.86) ***
Distance to health service		
Big problem	1 (Ref)	1 (Ref)
Not a big problem	0.93 (0.88–0.99) *	0.58 (0.55–0.61) ***
Use of contraceptive		
Not Using	1 (Ref)	1 (Ref)
Using	0.92 (0.86–0.98) *	0.67 (0.62–0.72) ***
Has health insurance		
No	1 (Ref)	1 (Ref)
Yes	0.92 (0.79–1.06)	0.68 (0.55.0.85) **
Exposure to Media		
No	1 (Ref)	1 (Ref)
Yes	0.95 (0.89–1.00) *	0.68 (0.65–0.71) ***

*** *p* < 0.001; ** *p* < 0.01; * *p* < 0.05.
